# Supplementary material for: Gut microbiome associations with acute malnutrition relapse in South Sudan
Source: Microbiol Spectr. 2026 Apr 27;14(6):e03587-25. doi: 10.1128/spectrum.03587-25 (PMC13228048; doi:10.1128/spectrum.03587-25)
Supplement: Supplemental figures — Fig. S1–S17. [file spectrum.03587-25-s0004.pdf]

Supplemental Figure 1

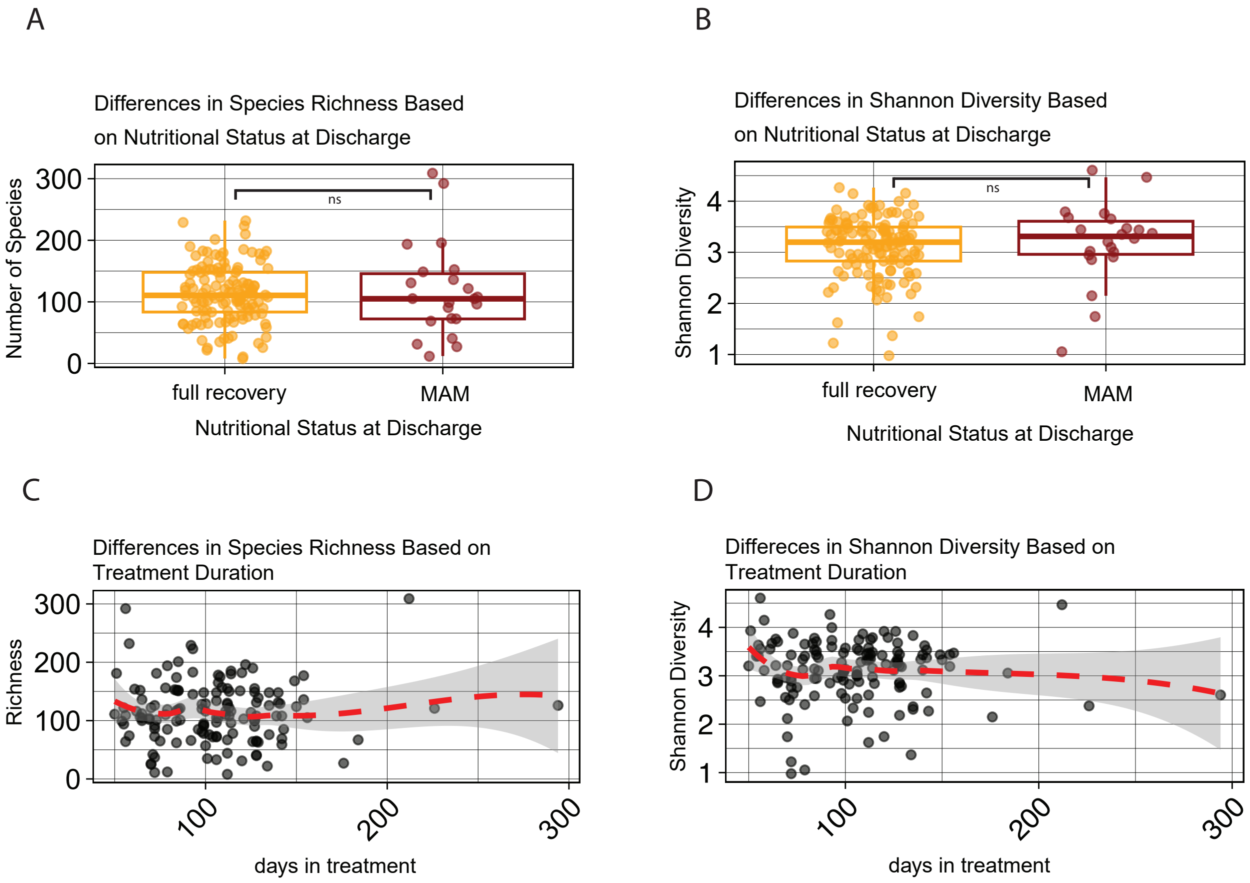

Supplementary Figure 1. Shannon diversity and richness associations with recovery according to WHO standards at discharge and time in CMAM treatment. (A) Species richness in the microbiomes at discharge for children discharged fully recovered or with MAM. Mann-Whitney with BH correction (full recovery: 110.5, MAM: 105.0, p = 0.79). (B) Shannon diversity in the microbiomes at discharge for children discharged fully recovered or with MAM. Mann-Whitney with BH correction (full recovery: 3.198, MAM:3.311, p = 0.43). (C) Species richness in the microbiomes at discharge for children with different lengths of days spent in CMAM treatment. Linear regression: (Adjusted r^2: -0.0061, p = 0.70). (D) Shannon diversity in the microbiomes at discharge for children with different lengths of days spent in CMAM treatment. Linear regression: (Adjusted r^2: -0.0056, p = 0.64)

Supplementary Figure 2

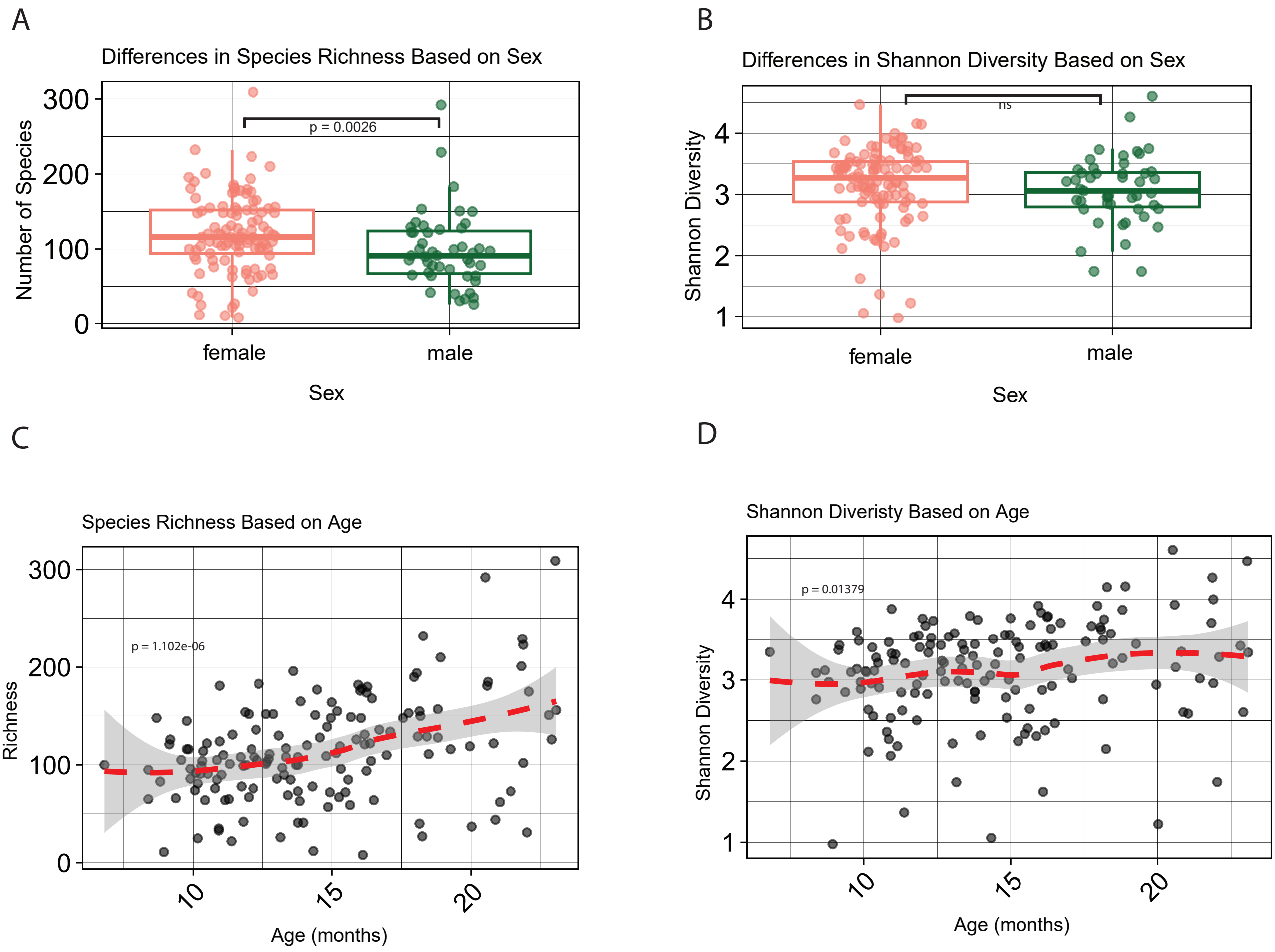

Supplementary Figure 2. Shannon diversity and richness associations with sex and age. (A) Species richness in the microbiomes of male and female children at discharge. Mann-Whitney with BH correction (male: 91, female: 116, p = 0.0026). (B) Shannon diversity in the microbiomes of male and female children at discharge. Mann-Whitney with BH correction (male: 3.059, female: 3.271, p = 0.13). (C) Species richness in the microbiomes of children at discharge versus age. Linear regression (Adjusted r^2: 0.1411, p = 1.102e-06). (D) Shannon diversity in the microbiomes of children at discharge versus age. Linear regression (Adjusted r^2: 0.03335, p = 0.01379)

Supplemental Figure 3

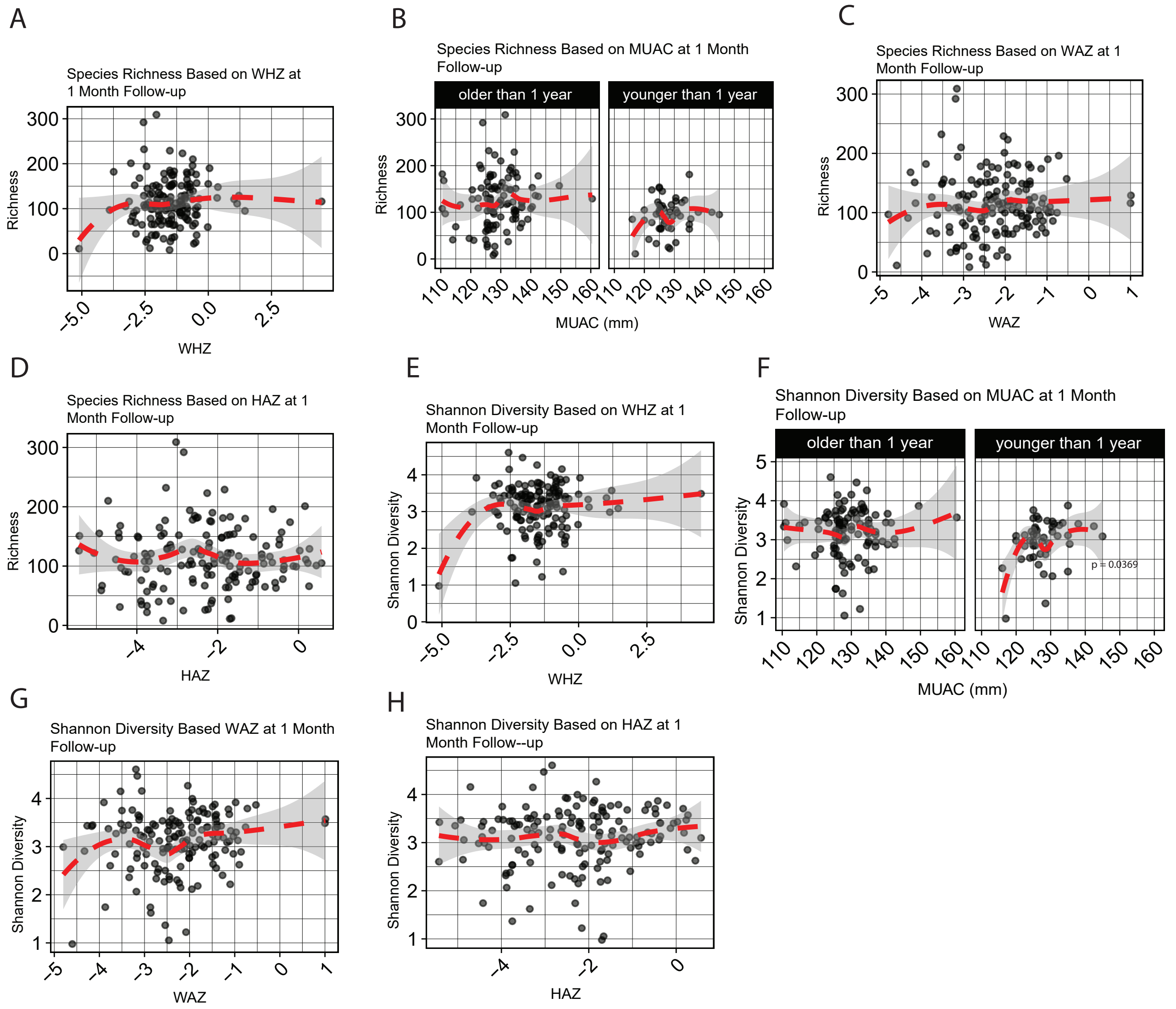

Supplementary Figure 3. Shannon diversity and richness associations with anthropometric measurements at 1 month follow-up. (A) Species richness in the microbiomes of children at discharge versus WHZ at 1 month follow-up. Linear regression (Adjusted r^2: -0.0005678, p = 0.3405). (B) Species richness in the microbiomes of children at discharge versus MUAC at 1 month follow-up stratified by age (less than 1 year or greater or equal to 1 year at time of sampling). Linear regression (greater or equal to 1 year: Adjusted r^2: -0.0002831, p = 0.3268, less than 1 year: Adjusted r^2: 0.005394, p = 0.2684). (C) Species richness in the microbiomes of children at discharge versus WAZ at 1 month follow-up. Linear regression (Adjusted r^2: -0.002737, p =0.4445). (D) Species richness in the microbiomes of children at discharge versus HAZ at 1 month follow-up. Linear regression (Adjusted r^2: -0.006651, p = 0.9617). (E) Species Shannon diversity in the microbiomes of children at discharge versus WHZ at 1 month follow-up. Linear regression (Adjusted r2: -0.001387, p = 0.2731). (F) Species Shannon diversity in the microbiomes of children at discharge versus MUAC at 1 month follow-up stratified by age (less than 1 year or greater or equal to 1 year at time of sampling). Linear regression (greater or equal to 1 year: Adjusted r^2: -0.00872, p = 0.7413, less than 1 year: Adjusted r^2: 0.07151, p = 0.0369). (G) Species Shannon diversity in the microbiomes of children at discharge versus WAZ at 1 month follow-up. Linear regression (Adjusted r^2: 0.01504, p = 0.07104). (H) Species Shannon diversity in the microbiomes of children at discharge versus HAZ at 1 month follow-up. Linear regression (Adjusted r^2: 0.001418, p = 0.2722).

Supplementary Figure 4

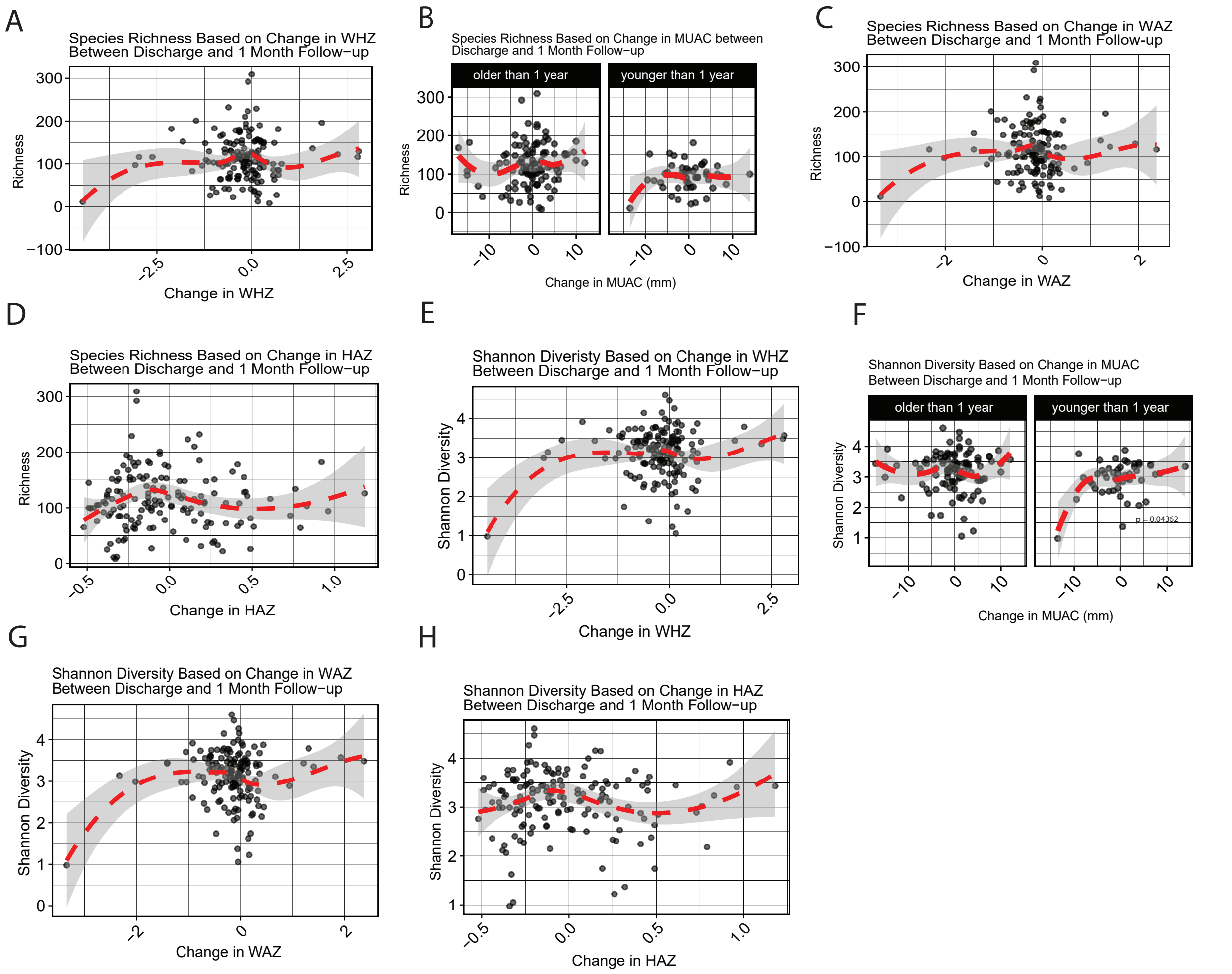

Supplementary Figure 4. Shannon diversity and richness associations with changes in anthropometric measurements between discharge and 1 month follow-up. (A) Species richness in the microbiomes of children at discharge versus changes in WHZ between discharge and 1 month follow-up. Linear regression (Adjusted r^2: -0.001908, p = 0.4). (B) Species richness in the microbiomes of children at discharge versus changes in MUAC between discharge and 1 month follow-up stratified by age (less than 1 year or greater or equal to 1 year at time of sampling). Linear regression (greater or equal to 1 year: Adjusted r^2: 0.001585, p = 0.2833, less than 1 year: Adjusted r^2: -0.01414, p = 0.56). (C) Species richness in the microbiomes of children at discharge versus changes in WAZ between discharge and 1 month follow-up. Linear regression (Adjusted r^2: -0.003941, p = 0.5244). (D) Species richness in the microbiomes of children at discharge versus changes in HAZ between discharge and 1 month follow-up. Linear regression (Adjusted r^2: -0.006328, p = 0.8225). (E) Species Shannon diversity in the microbiomes of children at discharge versus changes in WHZ between discharge and 1 month follow-up. Linear regression (Adjusted r^2: 0.004668, p = 0.1932). (F) Species Shannon diversity in the microbiomes of children at discharge versus changes in MUAC between discharge and 1 month follow-up stratified by age (less than 1 year or greater or equal to 1 year at time of sampling). Linear regression (greater or equal to 1 year: Adjusted r^2: -0.009528, p = 0.8678, less than 1 year: Adjusted r^2: 0.0657, p = 0.04362). (G) Species Shannon diversity in the microbiomes of children at discharge versus changes in WAZ between discharge and 1 month follow-up. Linear regression (Adjusted r^2: 0.002226, p = 0.2494). (H) Species Shannon diversity in the microbiomes of children at discharge versus changes in HAZ between discharge and 1 month follow-up. Linear regression (Adjusted r^2: -0.005812, p = 0.7216).

Supplementary Figure 5

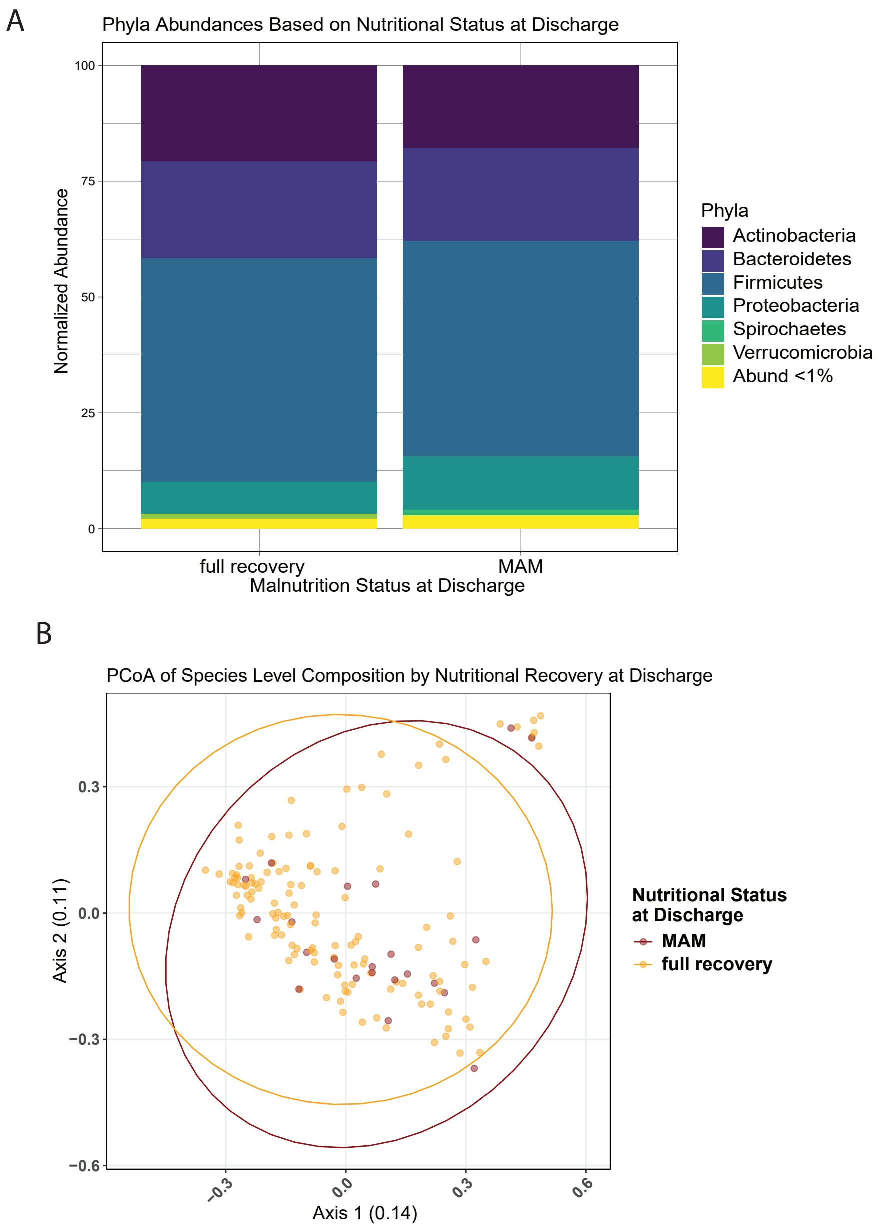

Supplementary Figure 5. Phyla and species distribution within microbiomes of children discharged fully recovered without AM or with MAM according to WHO standards. (A) Normalized abundance of phyla distribution in the microbiomes at discharge of children discharged fully recovered or with MAM. MaAsLin2 (Firmicutes p = 0.8191, Proteobacteria p = 0.04369, q = 0.4139, Bacteroidetes p = 0.8633, Verrucomicrobia p = 0.9955, Actinobacteria p = 0.9326, Spirochaetes p = 0.5169,. (B) PCoA of species distribution in the microbiomes at discharge of children discharged fully recovered or with MAM. Bray-Curtis dissimilarity PERMANOVA (F = 1.3643, Pr (>F) = 0.113). The 2 PCoA axes explain 14% and 11% of the variation in microbiome species distribution among samples respectively.

Supplementary Figure 6

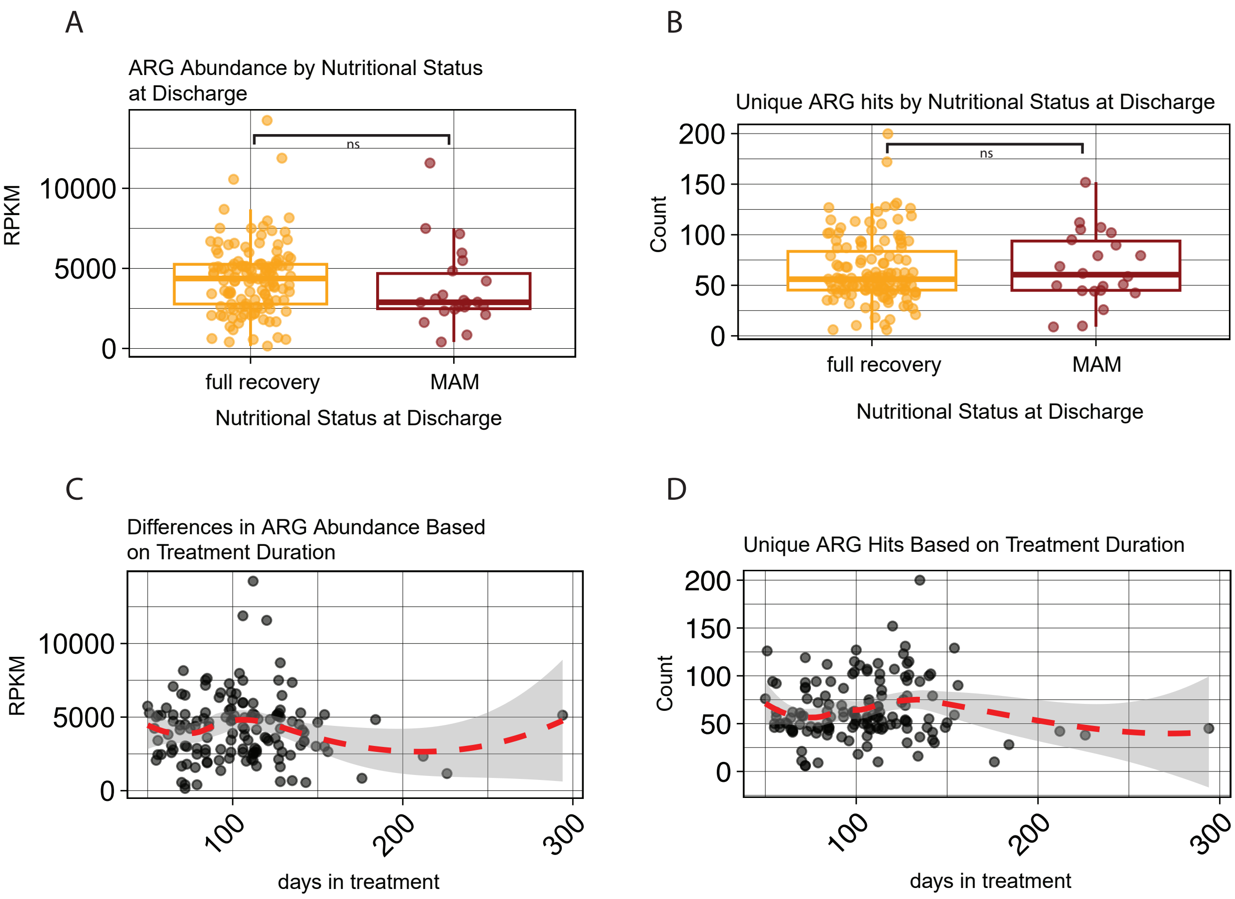

Supplementary Figure 6. Total and unique ARG abundance in the microbiomes at discharge of children discharged fully recovered without AM or with AM according to WHO standards. (A) ARG abundance in RPKM in the microbiomes at discharge of children discharged fully recovered or with MAM. Mann-Whitney with BH correction (fully recovered: 4369, MAM: 2884, p = 0.097). (B) Unique ARG hits in counts in the microbiomes at discharge of children discharged fully recovered or with MAM. Mann-Whitney with BH correction (fully recovered: 56.0, MAM: 60.5, p = 0.7). (C) ARG abundance in RPKM in the microbiomes at discharge for children with different lengths of days spent in CMAM treatment. Linear regression: (Adjusted r^2: -0.0059, p = 0.67). (D) Unique ARG hits in counts in the microbiomes at discharge of children with different lengths of days spent in CMAM treatment. Linear regression: (Adjusted r^2: -0.0060, p = 0.69).

Supplementary Figure 7

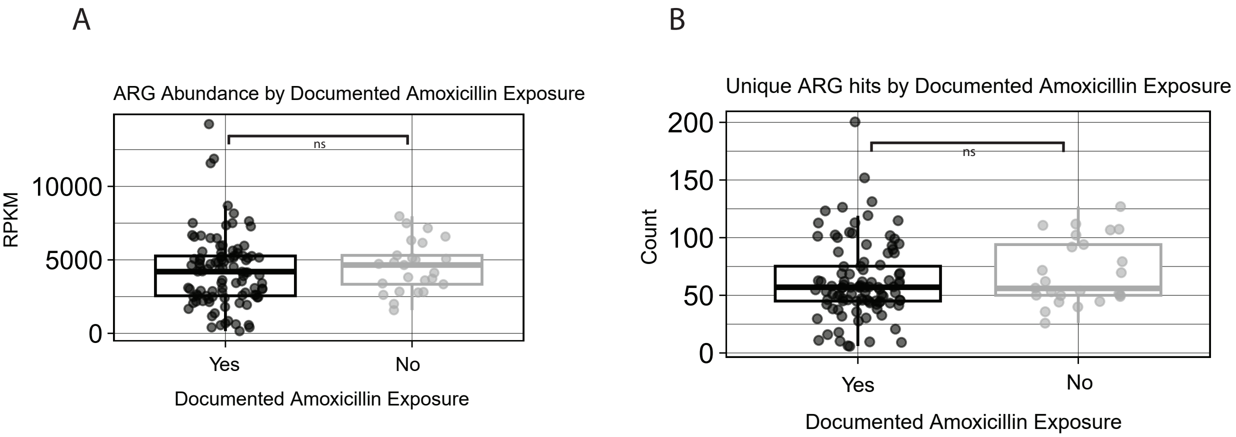

Supplementary Figure 7. Total and unique ARG abundance in the microbiomes at discharge of children exposed and not exposed to amoxicillin during treatment. (A) ARG abundance in RPKM in the microbiomes at discharge exposed or not exposed to amx. Mann-Whitney with BH correction (exposed: 4120, non-exposed: 4650, p = 0.36). (B) Unique ARG counts in the microbiomes at discharge exposed or not exposed to amoxicillin. Mann-Whitney with BH correction (exposed: 57, non-exposed: 56, p = 0.27).

Supplementary Figure 8

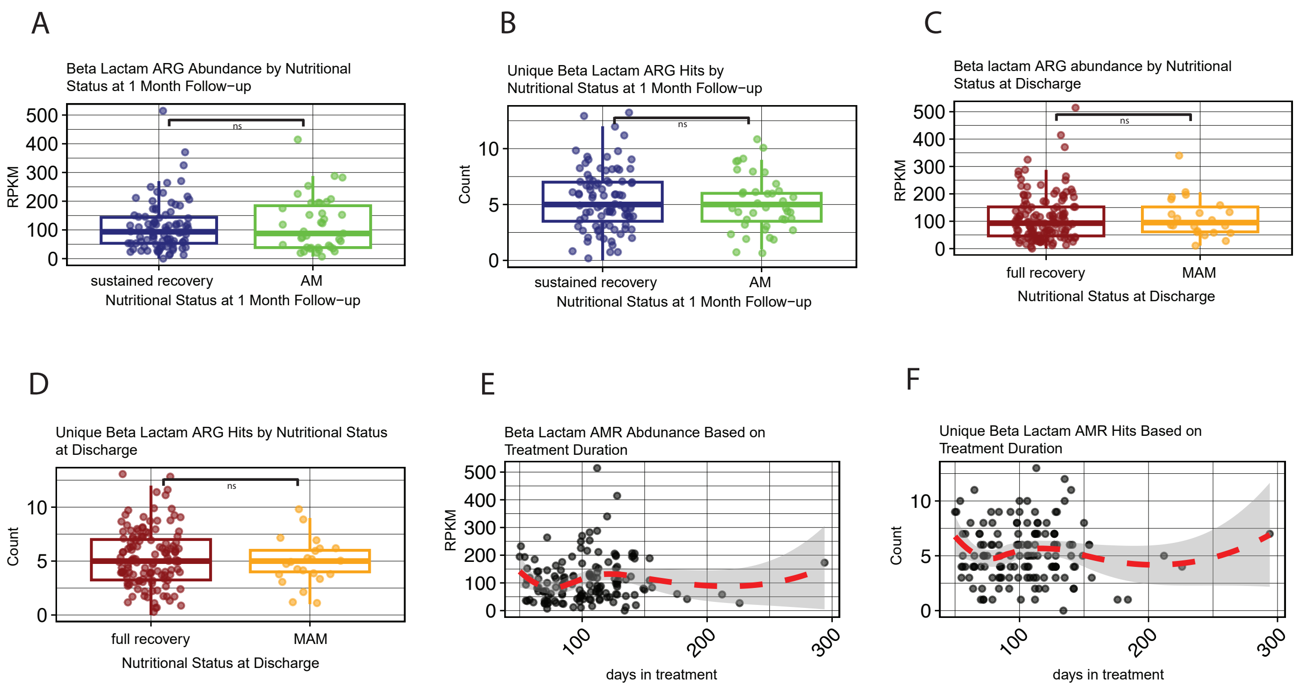

Supplementary Figure 8. Total and unique beta lactam ARG abundance in the microbiomes at discharge of children with different nutritional statuses at discharge and follow-up according to WHO standards. (A) Beta lactam ARG abundance in RPKM in the microbiomes at discharge of children who sustain recovery or relapse to acute malnutrition at 1 month follow-up. Mann-Whitney with BH correction (relapse: 87.64, sustain recovery: 93.52, p = 0.91). (B) Unique beta lactam ARGs in counts in the microbiomes at discharge of children who sustain recovery or relapse to acute malnutrition at 1 month follow-up. Mann-Whitney with BH correction (relapse: 5, sustain recovery: 5, p = 0.54). (C) Beta lactam ARG abundance in RPKM in the microbiomes at discharge of children discharged fully recovered or with MAM. Mann-Whitney with BH correction (fully recovered: 92.97, MAM: 95.08, p = 0.56). (D) Unique beta lactam ARGs in counts in the microbiomes of children discharged fully recovered or with MAM. Mann-Whitney with BH correction (fully recovered: 5, MAM: 5, p = 0.44). (E) Beta lactam ARG abundance in RPKM in the microbiomes at discharge of children with different lengths of stay in CMAM treatment. Linear regression: (Adjusted r^2: -0.0017, p = 0.39). (F) Unique beta lactam ARGs in counts in the microbiomes of children at discharge of children with different lengths of stay in CMAM treatment. Linear regression: (Adjusted r^2: -0.0069, p = 0.86).

Supplementary Figure 9

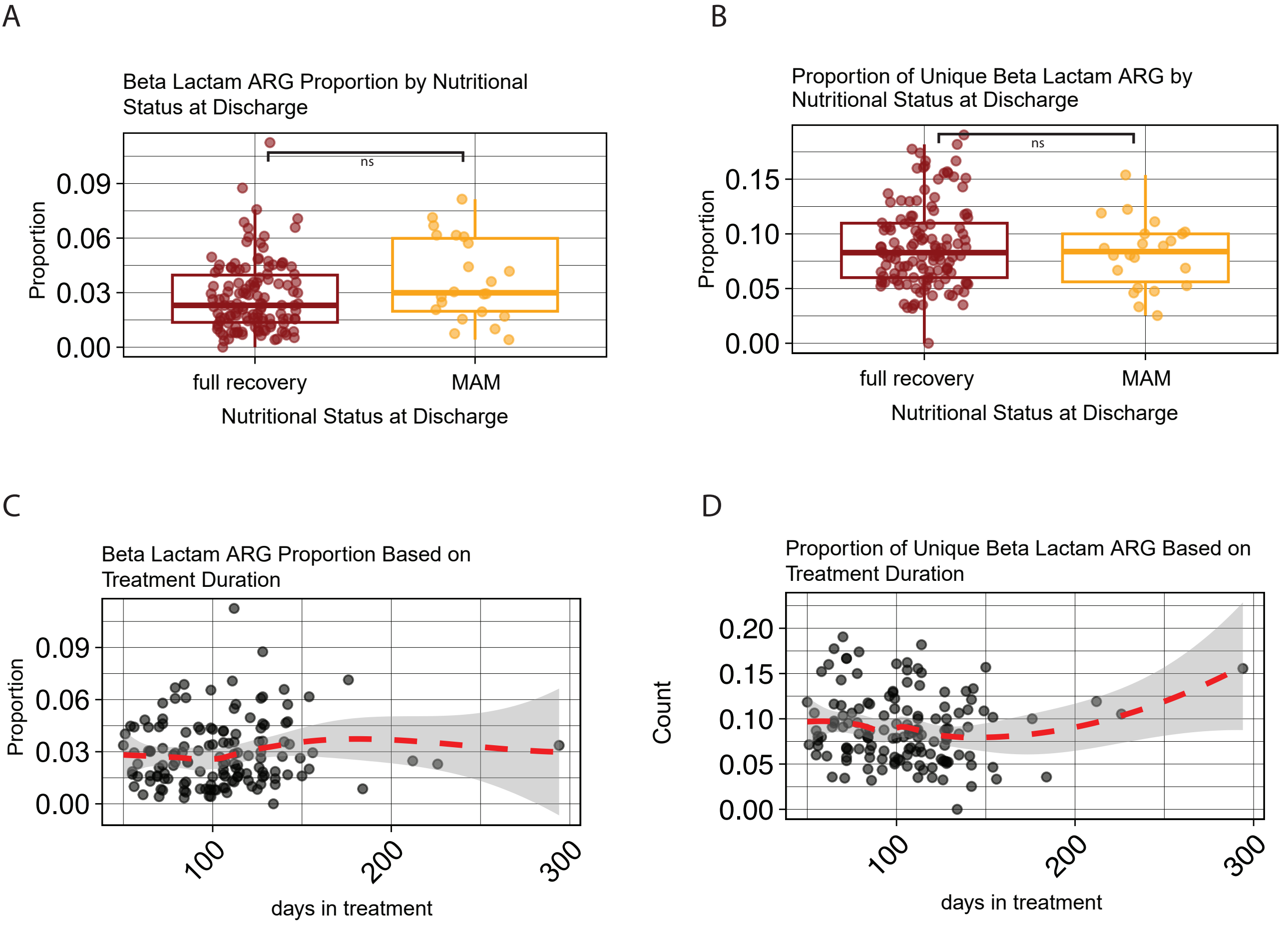

Supplementary Figure 9. Proportion of ARG gene content devoted to beta lactam ARGs in children discharged fully recovered without AM or with MAM according to WHO standards. (A) Proportion of gene content devoted to beta lactam ARGs in the microbiomes at discharge of children discharged fully recovered or with MAM. Mann-Whitney with BH correction (fully recovered: 0.02301, MAM: 0.02993, p = 0.069). (B) Proportion of unique genes that encode beta lactam resistance in the microbiomes at discharge of children discharged fully recovered or with MAM. Mann-Whitney with BH correction (fully recovered: 0.08284, MAM: 0.08380, p = 0.59). (C) Proportion of gene content devoted to beta lactam ARGs in the microbiomes of children with different lengths of days spent in CMAM treatment. Linear regression: (Adjusted r^2: 0.00071, p = 0.30). (D) Proportion of unique genes that encode beta lactam resistance in the microbiomes at discharge of children with different lengths of days spent in CMAM treatment. Linear regression: (Adjusted r^2: -0.0054, p = 0.62).

Supplementary Figure 10

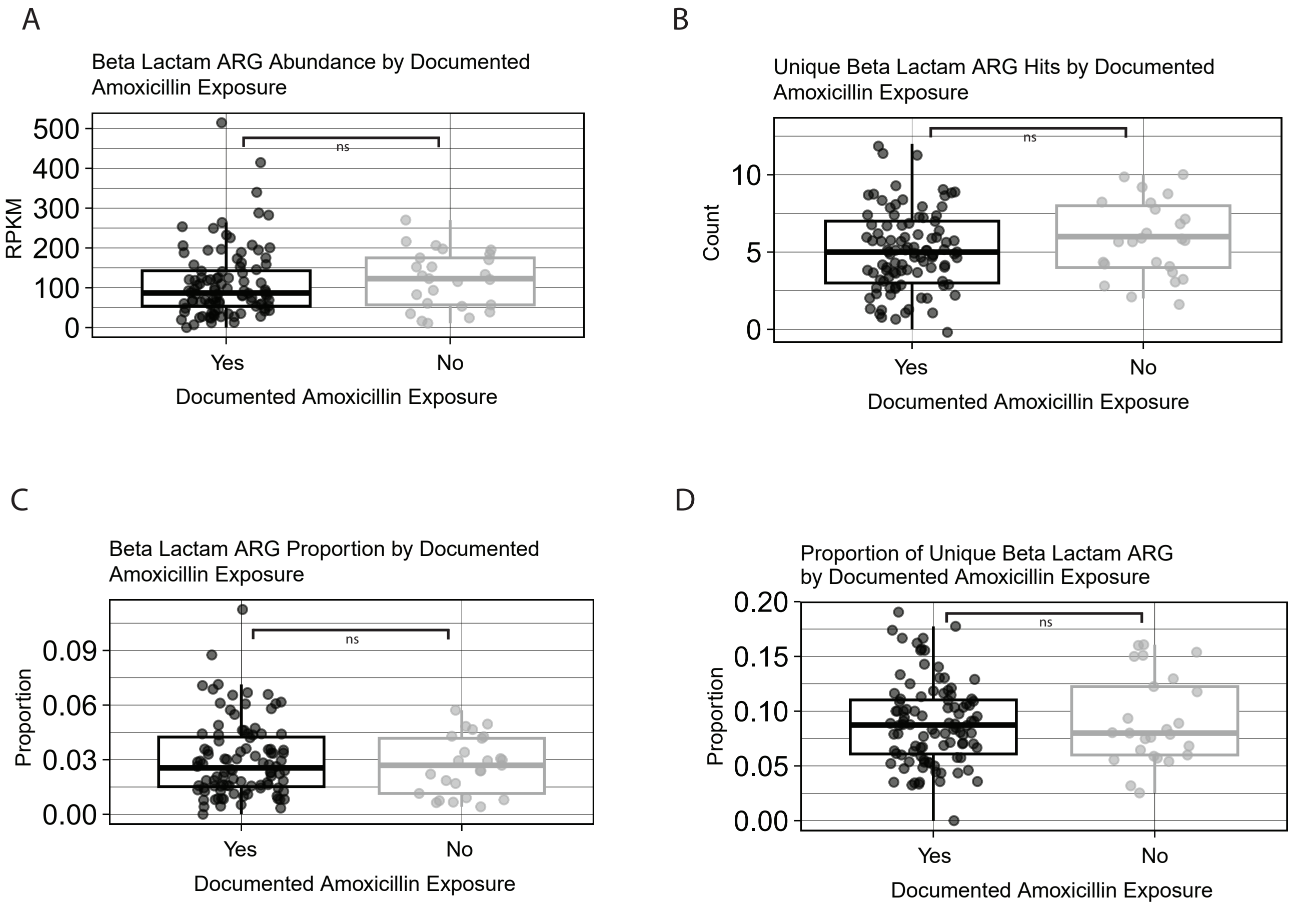

Supplementary Figure 10. Beta lactam genes in the microbiome and associations with amoxicillin exposure. (A) Beta lactam ARG abundance in RPKM in the microbiomes at discharge of children exposed or not exposed to amx. Mann-Whitney with BH correction (exposed: 86.88, non-exposed: 122.8, p = 0.32). (B) Unique beta lactam ARGs in counts in the microbiomes at discharge of children exposed or not exposed to amx. Mann-Whitney with BH correction (exposed: 5, non-exposed: 6, p = 0.19). (C) Proportion of gene content devoted to beta lactam ARGs in the microbiomes at discharge of children exposed or not exposed to AMX. Mann-Whitney with BH correction (exposed: 0.02550, non-exposed: 0.02693, p = 0.65). (D) Proportion of unique genes that encode beta lactam resistance in the microbiomes at discharge of children exposed or not exposed to amx. Mann-Whitney with BH correction (exposed: 0.08734, non-exposed: 0.08000, p = 0.96).

Supplementary Figure 11

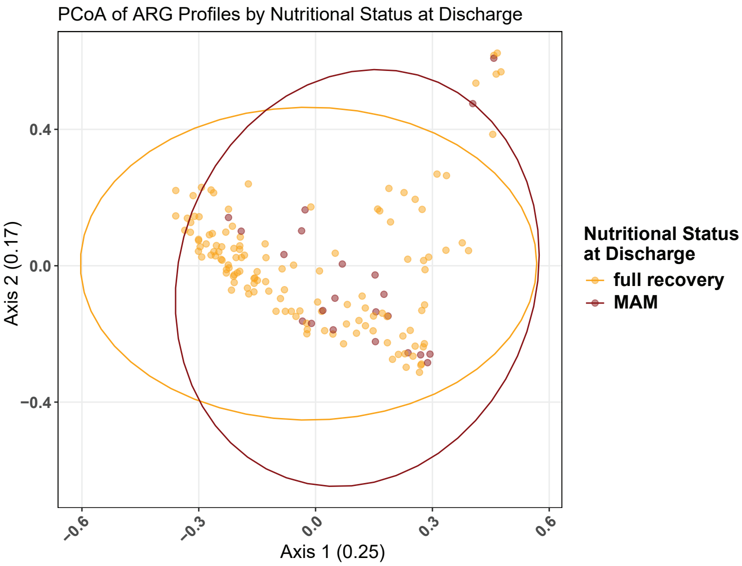

Supplementary Figure 11. Distribution of ARGs in the microbiome at discharge of children discharged fully recovered without AM or with MAM. PCoA of ARG distribution in the microbiomes at discharge of children discharged fully recovered or with MAM. Bray-Curtis dissimilarity PERMANOVA ($F = 2.33$, $Pr (>F) = 0.022$). The 2 PCoA axes explain 25% and 17% of the variation in microbiome ARG distribution among samples respectively.

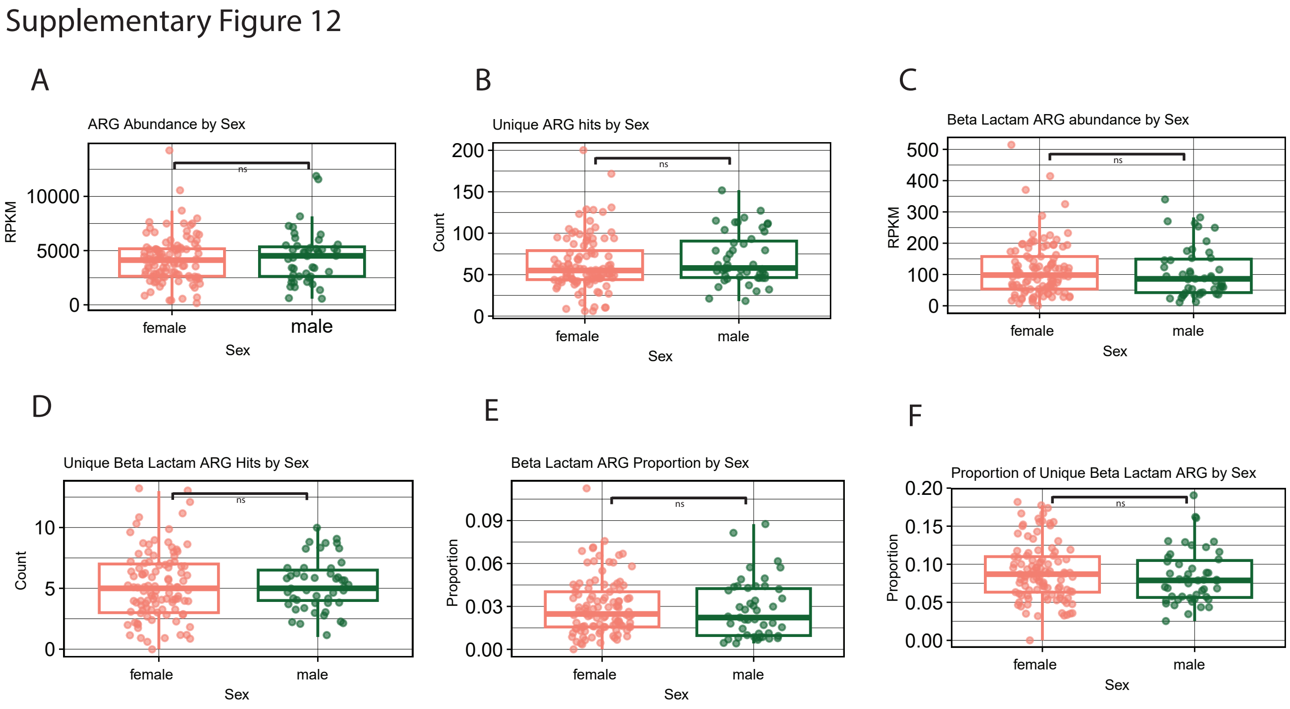

Supplementary Figure 12. ARGs in the microbiome at discharge of male and female children. (A) ARG abundance in RPKM in the microbiomes at discharge of male or female children. Mann-Whitney with BH correction (male: 4518, female: 4122, p = 0.67). (B) Unique ARG hits in counts in the microbiomes at discharge of male or female. Mann-Whitney with BH correction (male: 58, female: 55, p = 0.17). (C) Beta lactam ARG abundance in RPKM in the microbiomes at discharge of male or female children. Mann-Whitney with BH correction (male: 86.12, female: 98.17, p = 0.5). (D) Unique beta lactam ARGs in counts in the microbiomes at discharge of male or female children. Mann-Whitney with BH correction (male: 5, female: 5, p = 0.7). (E) Proportion of gene content devoted to beta lactam ARGs in the microbiomes at discharge of male or female children. Mann-Whitney with BH correction (male: 0.02222, female: 0.02472, p = 0.38). (F) Proportion of unique genes that encode beta lactam resistance in the microbiomes at discharge of male or female children. Mann-Whitney with BH correction (male: 0.07874, female: 0.08696, p = 0.25).

Supplementary Figure 13

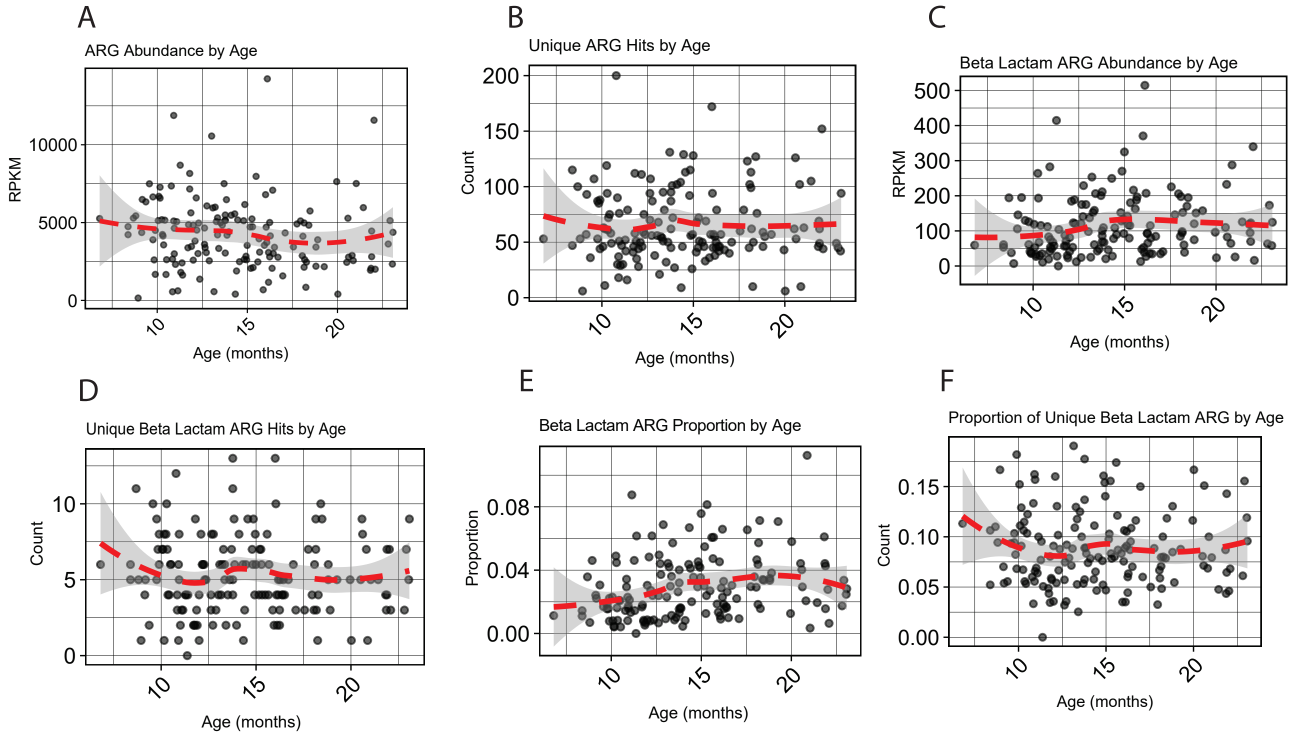

Supplementary Figure 13. ARGs in the microbiome at discharge and associations with age. (A) ARG abundance in RPKM in the microbiomes of children at discharge versus age. Linear regression (Adjusted r^2: 0.008462, p = 0.1324). (B) Unique ARG hits in counts in the microbiomes of children at discharge versus age. Linear regression (Adjusted r^2: -0.006327, p = 0.8223) (C) Beta lactam ARG abundance in RPKM in the microbiomes of children at discharge versus age. Linear regression (Adjusted r^2: 0.01422, p = 0.07664). (D) Unique beta lactam ARGs in counts in the microbiomes of children at discharge versus age. Linear regression (Adjusted r^2: -0.006025, p = 0.7575). (E) Proportion of gene content devoted to beta lactam ARGs in the microbiomes of children at discharge versus age. Linear regression (Adjusted r^2: 0.05717, p = 0.00175). (F) Proportion of unique genes that encode beta lactam resistance in the microbiomes of children at discharge versus age. Linear regression (Adjusted r^2: -0.006436, p = 0.8532).

Supplementary Figure 14

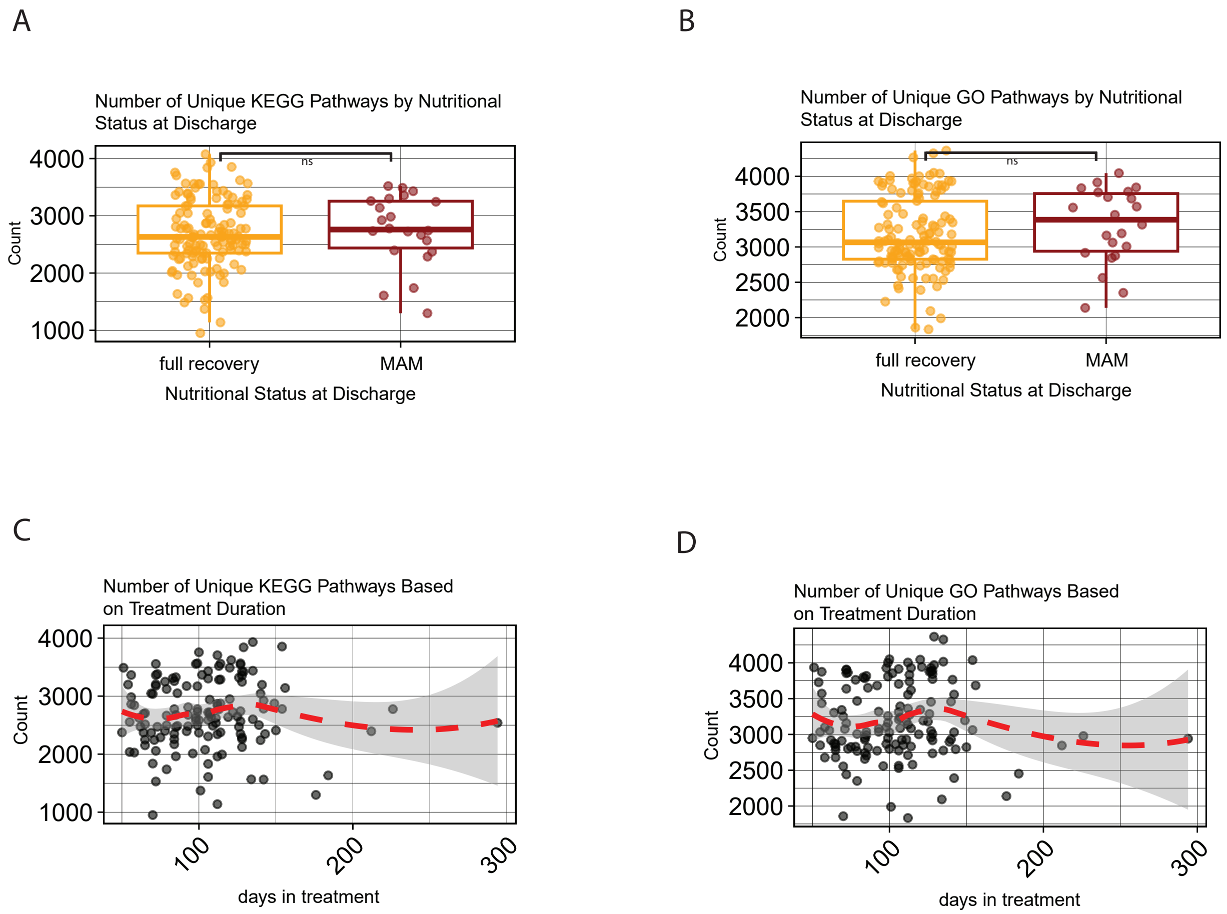

Supplementary Figure 14. Broad microbiome functional potential differences in children discharged fully recovered without AM or with MAM. (A) Unique KEGG pathways in counts in the microbiomes at discharge of children discharged fully recovered or with MAM. Mann-Whitney with BH correction (fully recovered: 2631, MAM: 2760, p = 0.37). (B) Unique GO pathways in counts in the microbiomes at discharge of children discharged fully recovered or with MAM. Mann-Whitney with BH correction (fully recovered: 3066.0, MAM: 3386, p = 0.27). (C) Unique KEGG pathways in counts in the microbiomes at discharge for children with different lengths of days spent in CMAM treatment. Linear regression: (Adjusted r^2: -0.0066, p = 0.78). (D) Unique GO pathways in counts in the microbiomes at discharge for children with different lengths of days spent in CMAM treatment. Linear regression: (Adjusted r^2: -0.0071, p = 0.97).

Supplementary Figure 15

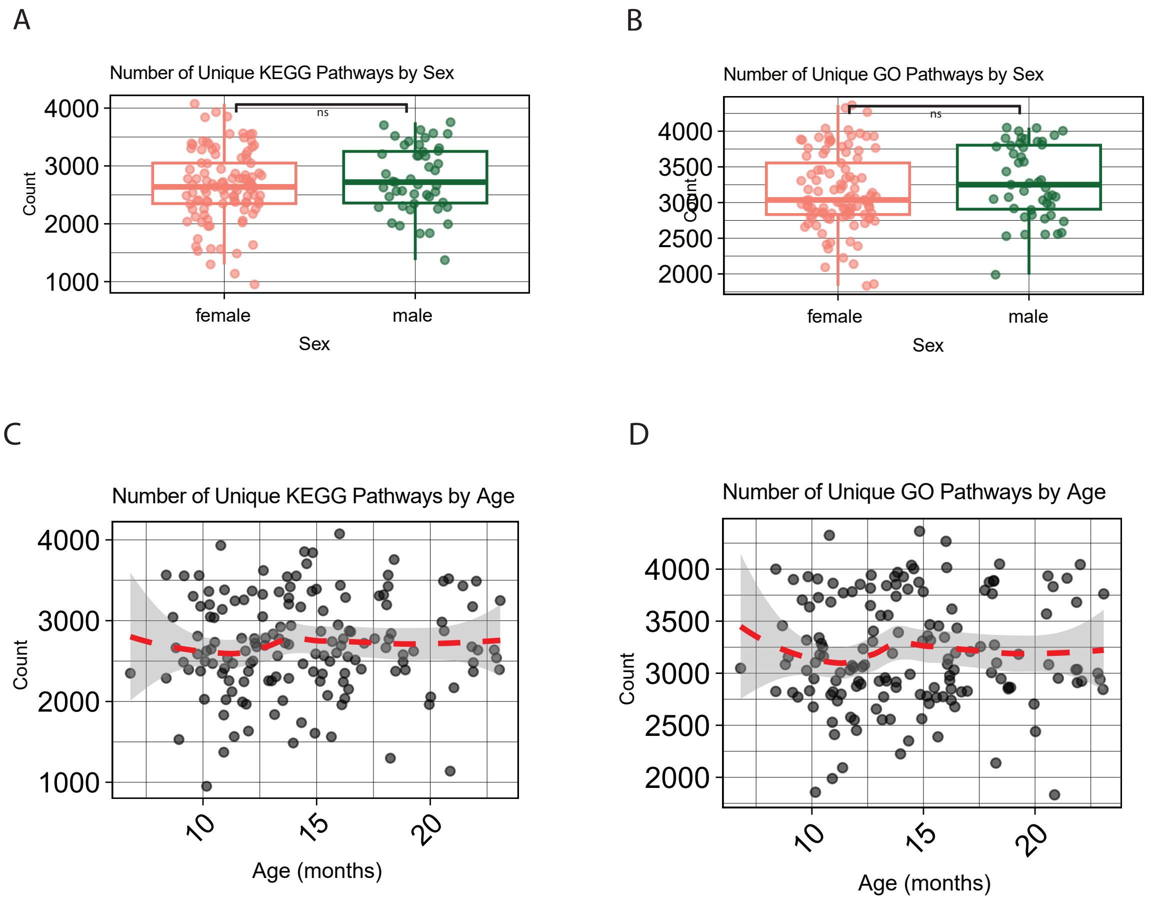

Supplementary Figure 15. Broad microbiome functional potential associations with age and sex. (A) Unique KEGG pathways in counts in the microbiomes at discharge of male or female children. Mann-Whitney with BH correction (male: 2720, female: 2638, p = 0.33). (B) Unique GO pathways in counts in the microbiomes at discharge of male or female children. Mann-Whitney with BH correction (male: 3251, female: 3036, p = 0.13). (C) Unique KEGG pathways in counts in the microbiomes of children at discharge versus age. Linear regression (Adjusted r^2: -0.003215, p = 0.4736). (D) Unique GO pathways in counts in the microbiomes of children at discharge versus age. Linear regression (Adjusted r^2: -0.005652, p = 0.6978).

Supplementary Figure 16

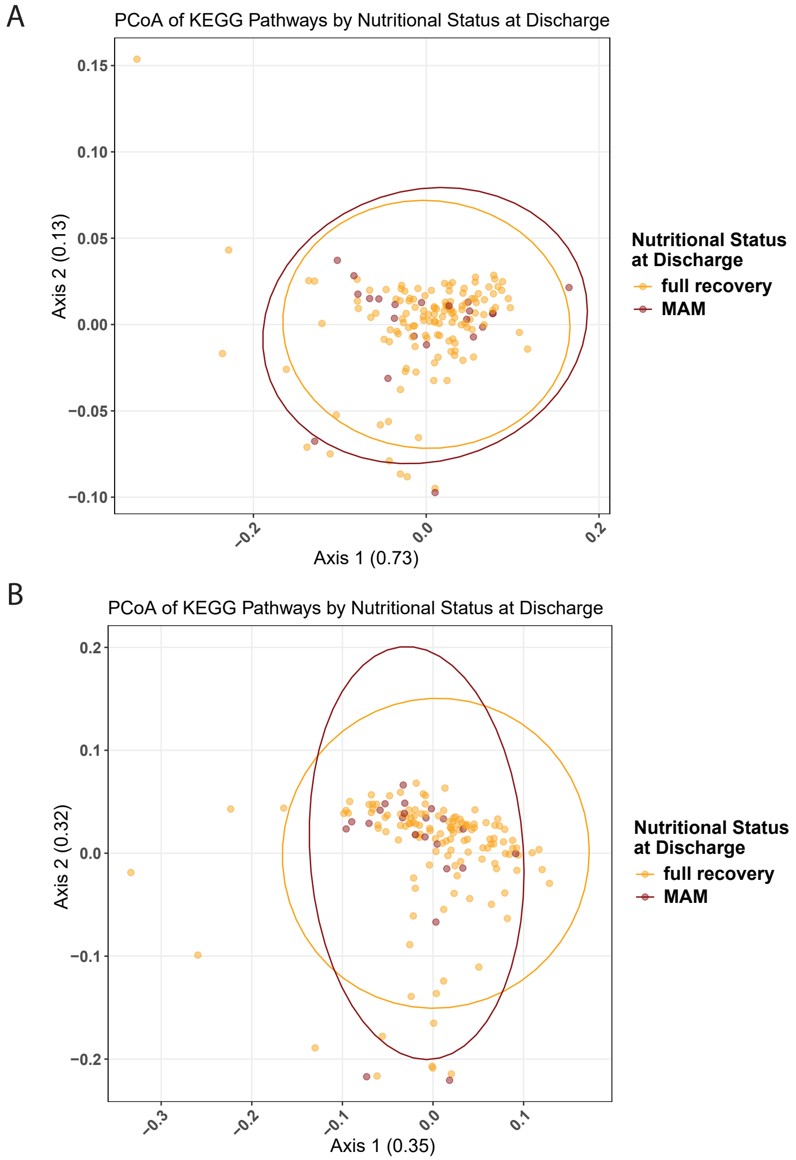

Supplementary Figure 16. Distribution of KEGG and GO pathways in the microbiomes at discharge of children discharged fully recovered without AM or with MAM. (A) PCoA of KEGG pathway distribution in the microbiomes at discharge of children discharged fulliy recovered or with MAM. PERMANOVA (F = 0.3205, Pr (>F) = 0.765). The 2 PCoA axes explain 73% and 13% of the variation in microbiome functional potential defined by KEGG among samples respectively. (B) PCoA of GO pathway distribution in the microbiomes at discharge of children discharged fully recovered or with MAM. PERMANOVA (F = 0.9085, Pr (>F) = 0.46). The 2 PCoA axes explain 35% and 32% of the variation in microbiome functional potential defined by GO among samples respectively.

Supplementary Figure 17

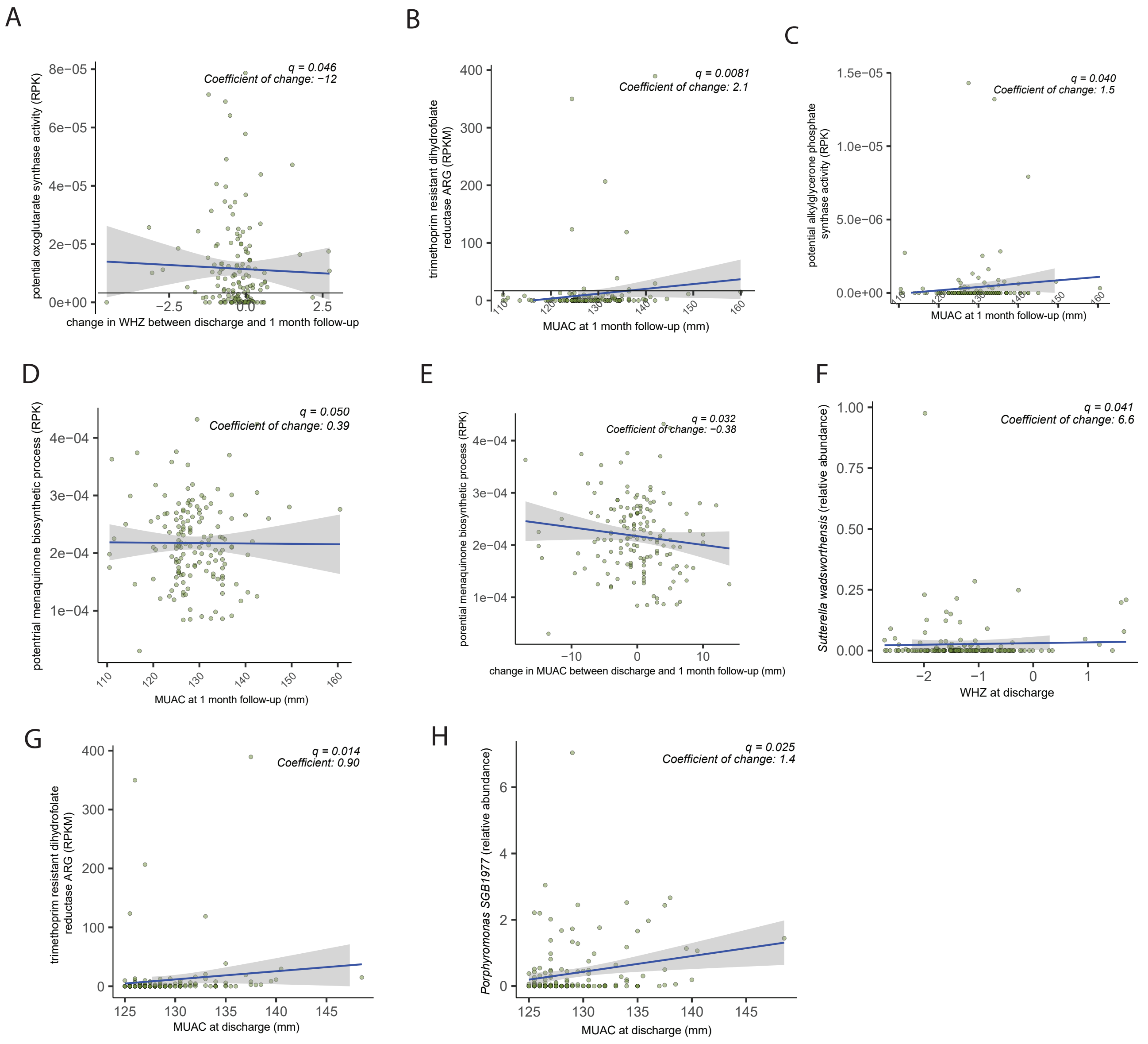

Supplementary Figure 17. Statistically significant coefficients of change of specific microbiome features at discharge in association with anthropometric measurements. (A) Change in oxoglutarate synthesis activity potential at discharge in reads per kilobase (RPK) against change in WHZ between discharge and 1 month follow-up (q = 0.046, coeff = -12). (B) Change in trimethoprim resistant dihydrofolate reductase ARGs at discharge in reads per kilobase million (RPMK) against MUAC at 1 month follow-up (q = 0.0081, coeff = 2.1). (C) Change in alkylglycerone phosphate synthase activity potential at discharge (RPK) against MUAC at 1 month follow-up (q = 0.040, coeff = 1.5). (D) Change in menaquinone biosynthetic process activity potential at discharge (RPK) against MUAC at 1 month follow-up (q = 0.050, coeff = 0.39). (E) Change in menaquinone biosynthetic process activity potential at discharge (RPK) against change in MUAC between discharge and 1 month follow-up (q = 0.032, coeff = -0.38). (F) Change in *Sutterella Wadsworthensis* relative abundance at discharge against WHZ at discharge (q = 0.041, coeff = 6.6). (G) Change in trimethoprim resistant dihydrofolate reductase ARGs at discharge (RPKM) against MUAC at discharge (q = 0.014, coeff = 0.90). (H) Change in *Porphyromonas SGB1977* relative abundance at discharge against MUAC at discharge (q = 0.025, coeff = 1.4).
